# Beyond a One-Time Scandal: Europe’s Onging Diesel Pollution Problem

**DOI:** 10.1289/ehp.124-A19

**Published:** 2016-01-01

**Authors:** Charles W. Schmidt

**Affiliations:** Charles W. Schmidt, MS, an award-winning science writer from Portland, ME, has written for *Discover Magazine*, *Science*, and *Nature Medicine*.

In September 2015 Volkswagen officials announced that nearly half a million diesel-powered cars sold by the company in the United States contained an illegal “defeat device” that disables pollution controls on the road. The admission came after the U.S. Environmental Protection Agency (EPA) determined that certain Volkswagen models complied with the federal emissions standard for nitrogen oxides (NO_x_) only under laboratory testing conditions.^1^ Depending on individual driving habits and road conditions, real-world NO_x_ emissions from affected 2.0-L engines could soar to 40 times the U.S. standard of 70 mg/mile, the EPA found. Similarly, emissions from the 3.0-L engines found in sport utility vehicles and larger cars could reach 9 times the standard.^2^ The scandal has since broadened to an estimated 11 million cars sold mostly in Europe by Volkswagen and subsidiaries Audi and Porsche.^3^

Diesel vehicles make up just 3% of the cars and pickup trucks driven in the United States, and those containing the illegal device make up less than half a percent of all cars, both diesel and gas-powered, says Allen Schaeffer, executive director of the nonprofit Diesel Technology Forum. From the perspective of health, emissions from cheating Volkswagen passenger vehicles might be too small to matter in the United States because there are so few of the vehicles, according to Gary Bishop, a research associate in the Department of Chemistry and Biochemistry at the University of Denver.

**Figure:**
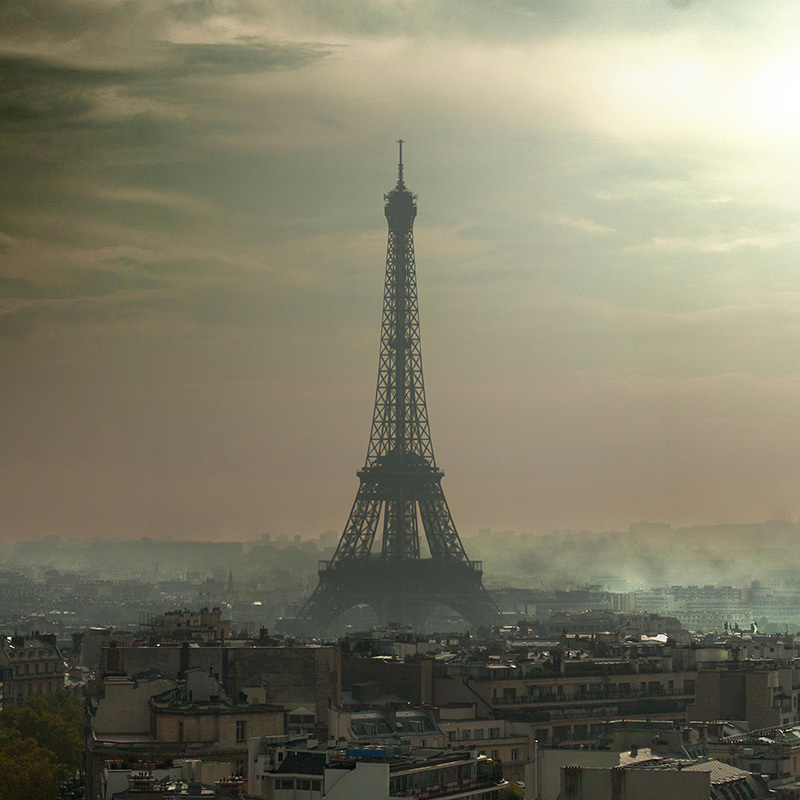
More than half the European passenger fleet is diesel-powered. Although the European Union has been progressively tightening vehicle emissions for decades, new diesel cars still produce on-road nitrogen oxide emissions that far exceed the current standard. Efforts to reduce diesel emissions would likely make the cars more costly, but experts say it can—and should—be done. © RooM the Agency/Alamy

By contrast, more than half of Europe’s passenger fleet is diesel-powered. The scandal therefore has had the added effect of spotlighting the persistent problem of NO_x_ pollution in Europe, where diesel emissions are a major contributor to poor urban air quality. To understand the potential health consequences of the emissions breach, however, one must first understand the risks associated with different components of diesel exhaust.

## A Stubborn NO_x_ Problem

NO_x,_ which diesel engines produce at high levels, is a collective term for gases including nitrogen oxide (NO) and nitrogen dioxide (NO_2_). NO has relatively minor health impacts at environmental levels. NO_2_, however, produces health effects ranging from mild cough and mucous membrane irritation to severe exacerbation of lung conditions such as chronic obstructive pulmonary disease and asthma.^4^

A November 2015 report by the European Environment Agency (EEA) estimated that 8–12% of Europe’s population is exposed to levels of NO_2_ that exceed the World Health Organization’s air quality guideline of 40 µg/m^3^.^4^ The highest levels were measured near highways, where diesel vehicles contribute about 80% of traffic-related NO_x_ emissions.^4^ Diesel exhaust is also associated with other air pollutants. Among them are ground-level ozone (O_3_), which forms when NO_2_ molecules interact with oxygen in the presence of sunlight, and fine sooty particulates measuring 2.5 µm or less (PM_2.5_) in the exhaust stream. These pollutants can travel deep into the lungs and elevate risks for DNA damage, heart attacks, and premature death.^5^^,^^6^^,^^7^

Urban NO_2_ exposures can have dire consequences, contributing to an estimated 75,000 premature deaths throughout the European continent in 2012. Ground-level O_3_ exposures, meanwhile, contributed to an estimated 17,000 premature deaths, and 432,000 premature deaths were attributed to PM_2.5_. But unlike NO_2_—which derives mainly from diesel emissions— O_3_ comes from a wide number of sources besides NO_2_–sunlight interaction. Similarly, PM_2.5_ is also released by agriculture, industrial facilities, home heating, and other processes.^8^

Belching clouds of exhaust, earlier diesel engines lacked the technologies that have lowered PM emissions by more than 90% since they came into widespread use in Europe and the United States during the 1990s.^9^ These advanced emission controls comprise what’s known as clean diesel technology, which also includes cleaner fuels.^10^ Schaeffer says clean diesel optimizes low emissions, fuel efficiency, and performance. Its emissions may also be less harmful than emissions from older diesel vehicles.

Animal studies of unfiltered diesel exhaust conducted prior to the emergence of this newer technology found evidence suggestive of carcinogenicity,^11^ and in 2012 diesel engine exhaust was listed as a Group 1 (i.e., confirmed) human carcinogen by the International Agency for Research on Cancer, based in part on occupational findings of lung cancer in exposed truck drivers and miners.^12^ Studies published in 2015, however, showed no evidence of carcinogenicity in rats exposed in the laboratory to diesel exhaust that meets the EPA’s more stringent 2007 and 2010 emissions standards, suggesting it may pose a lesser cancer risk to humans. ^11^^,^^13^^,^^14^

However, these same studies found evidence of abnormalities other than tumors, including mild lung inflammation, oxidative stress, decreased pulmonary function, and histological changes at the highest exposure levels. According to the authors, these effects were consistent with effects observed previously in rats exposed chronically to NO_2_.^13^

**Why Are There So Many Diesel Cars in Europe?**

Europe’s rise in diesel cars is rooted in well-intentioned efforts by national governments to reduce carbon emissions from the transportation sector. Countries that signed the Kyoto Protocol agreed to mandated greenhouse gas reduction targets, which could be met in the manner of their choosing.^25^ Perceiving an opportunity to meet these targets relatively cheaply, EU countries incentivized the purchase of diesel cars with tax breaks on diesel fuel.^26^ Unlike gasoline-powered engines that combust fuel with a spark plug, diesel engines compress fuel until it undergoes a controlled explosion. They’re more efficient than gasoline- powered engines, says Allen Schaeffer of the Diesel Technology Forum, and the resulting fuel savings correlate with reduced emissions of carbon dioxide, the principal greenhouse gas implicated in climate change. Subsequently, the percentage of diesel cars in the European passenger fleet shot up, in some countries quadrupling over the next 14 years.^27^

**Figure:**
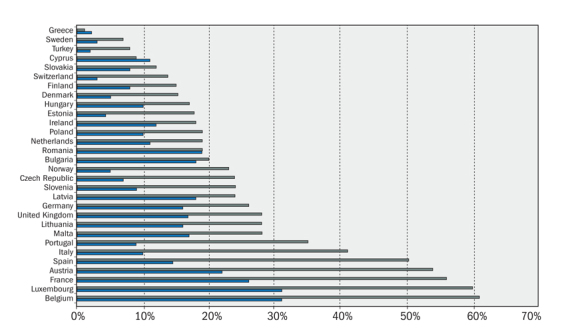

## Balancing PM and NO_2_

Assessing human health risks from diesel emissions on the basis of NO_x_ measures alone is challenging. That’s because European Union (EU) and U.S. NO_x_ standards don’t distinguish between the NO and NO_2_ constituents of emissions. Furthermore, depending on the NO_x_ emission control systems used, vehicles emit NO and NO_2_ in different proportions.

NO_x_ emissions from earlier diesel engines were dominated by NO, says S. Kent Hoekman, a research professor at the Desert Research Institute in Reno, Nevada. But catalytic emissions control systems adopted to meet increasingly stringent NO_x_ standards in the 1990s had the effect of altering this ratio: These systems tend to emit proportionately higher levels of NO_2_, especially when they don’t work correctly, even as they limit overall NO_x_ emissions.^15^ Multiple systems are engaged in selectively removing NO_x_ from exhaust streams, Hoekman says, and failure of any one system can lead to higher emissions.

As in the United States, EU regulators have traditionally relied on laboratory testing to evaluate whether diesel vehicles are meeting emissions reduction standards. Yet according to multiple sources interviewed for this story, EU regulators and automakers alike acknowledge that laboratory emissions and on-road emissions aren’t the same. Jens Borken-Kleefeld, a senior researcher with the International Institute for Applied Systems Analysis, explains that laboratory emissions and real-world emissions are instead assumed to be proportional to each other, such that mandated reductions in the laboratory will be met with comparable reductions on the road, although the absolute numbers won’t match. That’s true for both diesel and gasoline cars, he adds.

“This in itself would be satisfactory if real-driving and test-cycle emissions would decrease in tandem,” Borken-Kleefeld says. “But while test-cycle NO_x_ emissions have decreased by 80% since 1992, the real-driving NO_x_ emissions from diesel cars have actually increased by 20% over the same period.”^16^ The billion-dollar question, he says, is why such high on-road NO_x_ emissions have been allowed to persist for so long.

The EU has been progressively tightening vehicle emissions for decades, from its Euro 1 standard for passenger vehicles, which came into effect in 1991, through the Euro 6 standard for light passenger and commercial vehicles, adopted in 2014.^17^ But Borken-Kleefeld says that while PM emissions declined sharply as the standards became more stringent, real-world NO_x_ emissions never fell until the Euro 6 standard was adopted. Even now, he says, new diesel cars have real-world NO_x_ emissions that are 4–7 times higher than the current standard of 80 mg/km, although he points out that Euro 5 diesel cars produced, on average, twice that amount.

In the wake of the Volkswagen scandal, EU countries (and officials in the United States and elsewhere) have pledged to shift quickly from laboratory-based to roadside emissions testing, although important testing parameters are still being determined. The EU’s roadside testing regimen will phase in over the next few years, but until 2020 new diesel cars will still be allowed to emit NO_x_ at levels a little over twice the EU standard; after that, they may emit levels 50% higher than the standard.^18^ But Borken-Kleefeld says Europe could still eliminate almost all its NO_2_ exceedances by 2030 if diesel cars stay within the Euro limits and if other NO_x_ sources—for instance, trucks, power plants, and residential heating—continue to ratchet down their emissions.^19^

## The Current Situation in Europe

The Volkswagen scandal occurred in a year when air pollution in European cities was making headlines. In mid-March 2015 a dense smog cloud hung over cities in the United Kingdom, France, Spain, Germany, Italy, Poland, and Lithuania.^20^ The cloud comprised diesel exhaust pollutants and emissions from other sources, including ammonia-based particles generated from the application of manure fertilizers in agriculture, according to Julia Poliscanova, the clean vehicles and air quality officer for the European nonprofit Transport and Environment.For a brief period during that episode Parisian air pollution levels were worse than in any other city in the world, even Beijing and Delhi.^21^ Generally speaking, the country with the continent’s worst air pollution problem is Italy, where exposures to PM_2.5_, O_3_, and NO_2_ contributed to an estimated 59,500, 21,600, and 3,300 premature deaths, respectively, in 2012.^4^

Just how much early mortality can be blamed specifically on diesel pollution in Europe isn’t easy to gauge, however. According to the EEA, air quality in Europe overall is improving, even as PM_2.5_, ground-level O_3_, and NO_2_ levels associated with diesel exhaust remain stubbornly high.^4^ As noted previously, these pollutants have many sources, and their relative contributions to overall air pollution hinge on the complexities of atmospheric chemistry. For instance, NO in diesel exhaust reacts with O_3_ and destroys it at the tailpipe, which is why the highest O_3_ levels in Europe tend to be found in the countryside, whereas NO_2_ tends to peak in the urban core.^4^

As for the United States, a team of investigators from the Massachusetts Institute of Technology and Harvard University published an analysis suggesting that 59 people might die from the additional pollution emitted by Volkswagen’s cheating diesel vehicles. The authors attributed 87% of those deaths to PM_2.5_ formed when NO_x_ is converted to secondary ammonium nitrate particles in the atmosphere. The other 13% were attributed to the effects of ground-level O_3_, with none attributed specifically to NO_2._ However, the authors note a number of uncertainties in the models they used, which further research may help clarify.^22^

Meanwhile, in a growing trend, city officials throughout the EU are creating low-emission zones designed to keep older, more-polluting diesel and gasoline vehicles out of certain urban areas.^23^ Entry into the more than 70 low-emission zones established in Germany, for instance, depends on the color of a sticker mounted on the windshield. Red stickers denote old vehicles with more restricted access, while yellow and green stickers denote newer diesel- and gasoline-powered cars that can enter the zones more often.

Paris and London are following suit with similar approaches. On 27 September 2015 officials in Paris imposed the first-ever “car-free day” in 30% of the city between the hours of 11:00 a.m. and 6:00 p.m. The *Guardian* newspaper quoted one resident as saying, “The sky has never been this blue. It really is different without a hazy layer of pollution hanging in the air.”^24^

According to Borken-Kleefeld, however, low-emission zones have not been effective at reducing NO_2_ concentrations so far. That’s because virtually all new diesel cars that came on the market prior to Euro 6 emitted as much NO_x_ as previous generations, if not more. He predicts that only when new diesel cars with much lower emissions become widespread—within the next 10 years—or when gasoline-powered cars outnumber diesel cars will NO_2_ exceedances disappear.

Efforts to reduce NO_2_ emissions from passenger cars will make diesel technology more costly, says Poliscanova. This is especially true in the small market segment, she says, which will raise the attractiveness of cleaner gasoline hybrids.

But in the meantime, Poliscanova argues that urban air in much of Europe is barely fit to breathe, and diesel vehicles are the principal cause. “We’re not calling for the end of diesel,” she says. “But the technology must be clean if it is to be used in the future.”
